# Stable phase post-MI patients have elevated VEGF levels correlated with inflammation markers, but not with atherosclerotic burden

**DOI:** 10.1186/1471-2261-14-166

**Published:** 2014-11-22

**Authors:** Barbara Eržen, Mira Šilar, Mišo Šabovič

**Affiliations:** Department of Vascular Disease, University Clinical Centre, Ljubljana, 1000 Slovenia; Laboratory for Clinical Immunology & Molecular Genetics, University Clinic of Respiratory and Allergic Diseases, Golnik, Slovenia

**Keywords:** Vascular endothelial growth factor, Inflammation markers, Interleukin 6, Interleukin 8, Endothelial dysfunction, Myocardial infarction, Young adult patients

## Abstract

**Background:**

The role of vascular endothelial growth factor (VEGF) in patients in the stable phase after myocardial infarction (MI) has not yet been explored. Therefore, we compared the values of VEGF in post-MI patients with those obtained in healthy controls. Furthermore, we investigated whether the values of VEGF correlate to either inflammation markers or the atherosclerotic burden.

**Methods:**

41 male patients (on average 44 years old) in the stable phase after MI (on average 20.5 months after MI) were recruited, while 25 healthy age-matched males served as controls. Plasma levels of VEGF and several markers of inflammation were measured by standard procedures. The atherosclerotic burden was determined by the angiographic severity of coronary atherosclerosis, endothelial dysfunction (measured by ultrasound measurement of the flow mediated dilation of the brachial artery), the intima-media thickness of the common carotid artery and the ankle-brachial pressure index.

**Results:**

VEGF values were significantly elevated in post-MI patients compared to the controls (53.8 ± 42.7 pg/ml vs. 36.3 ± 8.9 pg/ml, p = 0.014). The elevated VEGF values significantly correlated to the (increased) values of the inflammatory molecules interleukin 6 and 8 (r = 0.37, p = 0.017; and r = 0.45, p = 0.003; respectively). In contrast, no correlation was found between VEGF and the parameters of the atherosclerotic burden, although FMD and IMT were significantly impaired in patients.

**Conclusions:**

We found that plasma levels of VEGF are increased in the stable phase after MI and correlate with inflammation cytokines, but not with the atherosclerotic burden. Thus, this suggests that increased levels of VEGF are a part of ongoing inflammatory activity. Since VEGF in these patients stimulates neovascularization of inflamed plaques and induces their destabilization, the VEGF level can have an important negative prognostic value. Clearly, further studies are needed to clarify the role of VEGF as a prognostic marker.

## Background

VEGF induces angiogenesis in several physiological conditions (for example embryogenesis), as well as in pathological states (for example tumour growth). VEGF induces angiogenesis through the formation of new vessels as a compensatory process in myocardial ischemia, but on the other hand, it can also be a mechanism producing destabilization of atherosclerotic plaques in coronary arteries [1–5]. Understanding of these evidently different roles of VEGF is still insufficient. Besides VEGF, several other angiogenic markers are known such as angiogenin, hepatocyte growth factor and basic fibroblast growth factor, though undoubtedly VEGF is the key angiogenic molecule. However, as mentioned above, its ability to assume either a pro-atherosclerotic or an anti-atherosclerotic role is somewhat confusing and still insufficiently explained.

There is only scare and conflicting data about the role of VEGF in coronary artery disease. Studies have shown that VEGF levels are increased in the acute phase of MI, as a response to myocardial ischemia. In acute MI patients who successfully underwent primary percutaneous intervention (PCI), VEGF levels reach their peak at day 7 [6–9], and return to normal after about 6 months [10]. There are conflicting data about the prognostic meaning of VEGF levels in the acute phase after MI. In a recently published Japanese prospective multicentre study it was shown that low levels of VEGF in the acute phase of MI are associated with a worse 6 months prognosis. In this study the incidence of recurrent acute coronary syndrome and stroke were significantly higher in the low VEGF group [6]. On the other hand, and in complete contrast, a European study showed that high VEGF levels in the acute phase of MI are related to an adverse outcome during a 6 months follow-up [11]. Discussing these conflicting results one could only conclude that VEGF may have an important prognostic value, but one which surprisingly may be positive or negative. From the clinical point of view it is even more important to investigate the role of VEGF in the stable phase of the disease.

Therefore the aim of the present study was to investigate whether VEGF is increased in the stable phase of coronary artery disease and to explore its role in this period. For this purpose we compared serum levels of VEGF in young post-MI patients and healthy age-matched controls. Furthermore, we were interested whether VEGF is related to the severity of inflammation (indicated by increased levels of serum inflammatory molecules) or to the degree of atherosclerotic burden. If associated with inflammation it could be deduced that VEGF probably has a negative angiogenic role in the inflammatory process inducing destabilization of plaques, whereas if associated with the atherosclerotic burden this suggests that it has a positive angiogenic role inducing angiogenesis in ischemic areas.

## Methods

### Study population

The study group consisted of 41 patients with coronary heart disease. In all patients MI was defined by a positive troponin level, ECG changes and obstructive coronary lesions confirmed by angiography. Inclusion criteria were male gender, age at the time of myocardial infarction less than 50 years, more than 6 months and less than 3 years from the time of MI to the time of the trial performance (an average of 20.5 months), and coronary artery disease in the stable period (absence of unstable AP). Exclusion criteria were malignancy, heart failure, an acute inflammation state 14 days before the study, a history of diabetes mellitus, anticoagulant therapy, and the use of any other drugs except ACE inhibitors, beta blockers, antiplatelet drugs and statins. We divided the patients into three groups (one, two or three vessel disease) according to the number of diseased vessels seen on the coronarography at the time of MI. Significant stenosis was determined as stenosis above 50%.

The control group consisted of 25 healthy male volunteers, who did not differ from the patients regarding age and had no manifested atherosclerotic disease.

All subjects were informed about the study protocol and gave their informed consent. The study protocol conformed to the ethical guidelines of the 1975 Declaration of Helsinki, and was approved by the State Ethics Committee of Slovenia.

### Clinical examination and laboratory procedures

One visit by the subjects was necessary at which their medical history was recorded, and a clinical examination, biochemical tests and ultrasound measurements were performed. All ultrasound measurements were performed in the morning. Patients were on their regular therapy and had taken their usual dose of morning medicine. Any smokers abstained from smoking on that day.

A detailed family and personal history was recorded, and a clinical examination performed according to a questionnaire. Systolic and diastolic blood pressures were measured with a mercury sphygmomanometer after a minimum of 10-min rest in the sitting position. The average of three measurements was taken. Anthropometric parameters were determined, and BMI was calculated as weight in kilograms divided by the square of the height in meters, and WHR was calculated as waist size divided by hip size.

Blood for laboratory analysis was collected in the morning after a 12-hour overnight fast. Samples were drawn from the antecubital vein. Blood for the analysis of glucose and lipids was collected without additives, centrifuged and samples of serum were analysed. Concentrations of glucose, total cholesterol, high density lipoprotein (HDL) cholesterol and triglycerides were determined by standard colorimetric assays from the fresh samples of serum (Ektachem 250 Analyzer, Eastman Kodak Company, Rochester, USA). Low density lipoprotein (LDL) cholesterol was calculated using Friedewald's formula. High sensitivity CRP (hsCRP) was measured with a fully automated, latex-enhanced nephelometric immunoassay (N High Sensitivity CRP, Dade Behring Marburg, Germany).

Concentrations of VEGF, angiogenin and interleukin-8 (IL-8were measured by Cytometric Bead Array (BD Biosciences Pharmingen, San Diego, CA, USA) containing microparticles dyed to different fluorescence intensities. The anaphylatoxin-captured beads were incubated with standards (purified from human plasma) or test samples followed by a wash and incubation with phycoerythrin-conjugated detection antibodies to form sandwich complexes. Flow cytometric analysis was performed using a FACSCalibur flow cytometer (Becton Dickinson Immunocytometry Systems, BDIS, BD Biosciences, San Jose, CA, USA). Data were acquired and analysed using the Beckton Dickinson Cytometric bead array (CBA) software.

Serum tumour necrosis factor- α (TNF-α) (Quantikine® HS Human TNF- α Immunoassay, R&D Systems, USA), interleukin-6 (IL-6) (Quantikine® Human IL-6 Immunoassay, R&D Systems, USA) and the serum cell adhesion molecules ICAM-1, VCAM-1, selectin-P, selectin-E (Parameter® ICAM-1, Parameter® VCAM-1, Parameter® selectin-P, Parameter® selectin-E, R&D Systems UK) were determined by enzyme-linked immunosorbent assays.

### Vascular studies

**a.) Measurement of endothelial dysfunction (ED);** Flow mediated (FMD) (endothelium-dependent) and glyceryl trinitrate (GTN)-induced dilation (endothelium-independent) of the right brachial artery were studied using a high-resolution B mode Diasonics VST ultrasound system with a 10 MHz linear array transducer. The subjects rested in the supine position for ten minutes before hemodynamic measurements were performed. The right brachial artery was scanned in the longitudinal section 2 to 15 cm above the elbow to find the clearest images of the anterior and posterior wall layers. The mean arterial diameter was measured at the end of the diastole, which was determined by simultaneous monitoring of the electrocardiogram (concurrent with onset of the QRS complex). At least three cardiac cycles were analysed for each scan and the measurements averaged. The flow velocity was measured at a fixed incident angle of 68° to the vessel with the range gate of 1.3 mm located in the centre of the artery. The baseline blood flow was estimated by multiplying the velocity time integral of the Doppler flow signal (corrected for incident angle) by the vessel cross-sectional area. Hyperemic flow increase was induced by inflation of a blood pressure tourniquet placed around the forearm to a pressure of 300 mmHg for 4.5 minutes. Hyperemic flow was recorded for the first 15 seconds and diameter measurements were taken 45–60 seconds after cuff deflation. The endothelium-dependent dilation was expressed as the percentage change of the diameter after reactive hyperaemia, relative to the baseline scan. Ten minutes were allowed for vessel recovery, after which a further resting scan was taken. A sublingual tablet of 0.5 mg of GTN was then administered, and 4.5 minutes later, the final scan was performed. The endothelium-independent dilation was expressed as the percentage change in diameter after GTN administration, relative to the baseline scan. The same investigator, who was blind to the subjects’ characteristics, carried out all measurements. To assess the reproducibility of the measurements, 40 subjects were selected randomly for repeated vascular studies. The correlation coefficient between the absolute differences and mean values of paired measurements was 0.92, p < 0.05.

**b.) Measurement of intima-media thickness;** IMT of the common carotid artery (CCA) was assessed by the B-mode ultrasound technique. Measurements of IMT were obtained from the far wall of the distal part of the CCA (immediately proximal to the carotid bulb) on both sides. All studies were performed on a single ultrasound machine (Diasonics VST ultrasound system) using a linear-array 10-MHz scan head with standardized image settings, including resolution mode, depth of field, gain, and transmit focus. All sonograms were obtained with the patient in the supine position and their head turned slightly to the contra lateral side. Each ultrasound examination was performed as an independent study, without any knowledge of the history of MI and risk factors. The IMT was measured as the distance from the leading edge of the near-field (intimal-luminal surface) and far-field (medial-adventitial) arterial wall. An average of three measurements on both sides was derived. The mean IMT was calculated as the average of the left and right CCA.

**c.) Measurement of ankle-brachial pressure index (ABPI);** the systolic blood pressure in both arms was taken with a blood pressure cuff and Doppler probe, after a minimum of 10-min rest in the sitting position, averaged, and divided into the systolic blood pressure in the posterior tibial or dorsalis pedis artery in the leg. The higher reading was used to determine the ABPI. ABPI was calculated separately for each leg. The mean ABPI was calculated as the average of the left and right leg.

### Statistical analysis

Variables showing a normal distribution, as determined by the Kolmogorov-Smirnov test, were expressed as means and standard deviations. Differences between the two groups were tested for significance by Student’s t-test for normally-distributed variables with equal variances, or by the Welch t-test for variables with unequal variances. Correlation between normally distributed variables was tested with the Pearson correlation coefficient. The criterion for statistical significance was a p value of less than 0.05. We constructed a multiple linear regression model to predict VEGF in patients on the basis of classical coronary risk factors, inflammatory parameters and functional and structural characteristics of the arterial wall. All calculations were performed by the IBM SPSS statistics 19 computer program.

A power analysis was performed to calculate the appropriate sample size to detect significant differences between the two groups at adequate power. All calculations, except the power analysis which was performed with the PASS 11 statistical program, were performed by the IBM SPSS statistics 19 computer program.

## Results

### Characteristics of the patients and controls at the time of trial performance

There were no differences in the age of patients and controls. BMI, but not WHR, was increased in post-MI patients in comparison to healthy controls. All patients were taking anti-platelet therapy, 19 (90%) statins (simvastatin or atorvastatin), 19 (90%) beta-blockers, and 19 (90%) patients ACE-inhibitors. Subjects in the control group were not taking any medication. Because of their medication, patients had significantly lower values of LDL cholesterol and lower systolic blood pressure in comparison to the controls. The characteristics of the patients and of the controls at the time of the trial performance are shown in Table 1.

**Table 1:** **Characteristics of the subjects and laboratory parameters at the time of the trial performance**

Parameter	Controls (N = 25)	Patients (N = 41)	p
Age (years)	42.2 (4.9)	44.8 (5.4)	ns
BMI (kg/m^2^)	25.4 (3.4)	27.3 (2.7)	0.02
Waist-hip ratio (rel.)	0.93 (0.04)	0.94 (0.05)	ns
Systolic blood pressure (mmHg)	133 (13)	125 (14)	0.02
Diastolic blood pressure (mmHg)	87 (11)	82 (10)	ns
Total cholesterol (mmol/l)	4.99 (0.78)	4.55 (1.0)	ns
LDL-cholesterol (mmol/l)	3.11 (0.73)	2.6 (0.87)	0.02
HDL-cholesterol (mmol/l)	1.18 (0.26)	1.02 (0.28)	0.02
TG (mmol/l)	1.50 (0.57)	2.18 (1.57)	0.04
Blood glucose (mmol/l)	5.4 (1.3)	5.9 (1.7)	ns
Smokers (N)	4 (16%)	11 (27%)	ns
Ex-smokers (N)	4 (16%)	23 (56%)	<0.01
Family history (N)	2 (8%)	14 (34%)	0.01

Values are shown as means and standard deviations; p-for the difference between the two groups, ns-not significant, N-number, BMI-body mass index, rel.-relative, LDL-low density cholesterol, HDL-high density cholesterol, TG-triglycerides.

### VEGF, angiogenin and inflammatory molecules in the patients and controls at the time of trial performance

**Figure 1:**
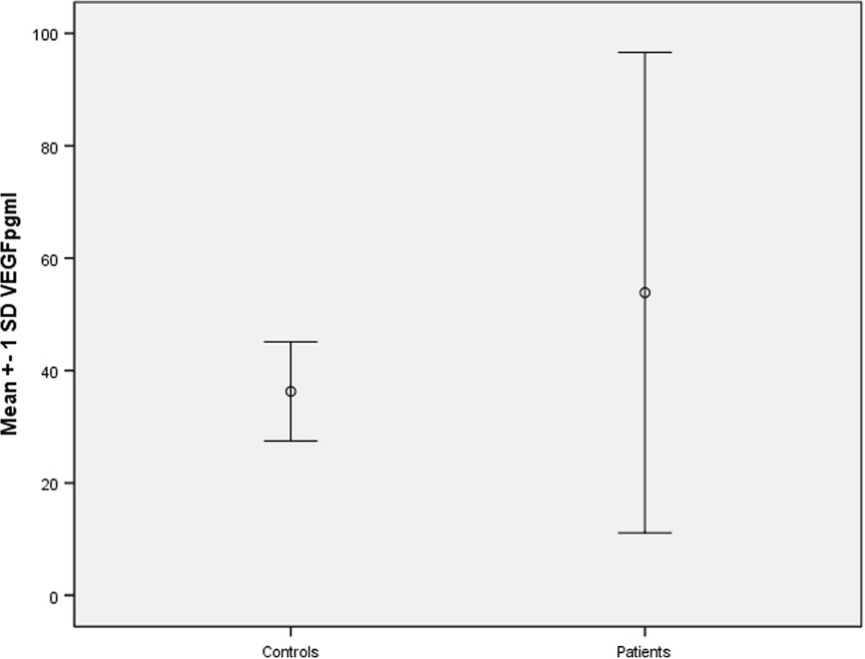
**VEGF in patients and controls.** *Legend: VEGF- vascular endothelial growth factor, SD-standard deviation.*

The patients had significantly higher levels of VEGF in comparison to the controls (53.8 ± 42.7 pg/ml vs. 36.3 ± 8.9 pg/ml, p = 0.014). The mean values of VEGF ± one standard deviation in patients and controls are shown in Figure 1. There were no statistically significant differences in angiogenin levels between the patients and controls (1.37 ± 0.46 pg/ml vs. 1.42 ± 0.38 pg/ml, p = 0.318).

The patients had significantly increased IL-6 and hsCRP in comparison to the controls. The values are shown in Table 2.

**Table 2:** **Angiogenic and inflammation markers of subjects at the time of the trial**

Parameter	Controls (N = 25)	Patients (N = 41)	p
VEGF (pg/ml)	36.3. ±8.9	53.8 ± 42.7	0.014
Angiogenin (pg/ml)	1.42 ± 0.38	1.37 ± 0.46	0.318
hs CRP (mg/l)	1.5 +/- 1.4	3.5 +/- 8.1	0.038
IL-6 (ng/ml)	2.5 ± 3.4	4.9 ± 4.1	0.016
IL-8	5.4 +/- 1.3	6.3+/- 3.0	0.102
IL-8 (pg/ml)	5.4 ± 1.3	6.3 ± 3.0	0.102
hs CRP (mg/ml)	1.5 ± 1.4	3.5 ± 8.1	0.038
VCAM-1 (ng/ml)	286.5 ± 60.1	283.3 ± 52.1	0.336
ICAM-1 (ng/ml)	182.3 ± 50.3	190.9 ± 44.9	0.775
Selectin-P (ng/ml)	52.9 ± 10.9	55.1 ± 13.4	0.373
Selectin-E (ng/ml)	44.7 ± 12.5	47.4 ± 14.8	0.112
TNF-α (ng/ml)	1.19 ± 1.12	0.93 ± 1.0	0.780

Values are shown as means and standard deviations; VEGF-vascular endothelial growth factor, IL-6 – interleukin 6, IL-8 – interleukin 8, hsCRP – high sensitive C-reactive protein, VCAM-1 – vascular cell adhesion molecule-1, ICAM-1 – intracellular adhesion molecule-1, TNF-α – tumour necrosis factor α.

### Vascular studies

In the patients both FMD and IMT were significantly increased in comparison to the controls. There were no significant differences in ABPI between the two groups. The data are shown in Table 3.

**Table 3:** **Vascular studies**

Parameter	Controls (N = 25)	Patients (N = 41)	p
FMD (%)	8.8 ± 6.5	5.5 ± 4.3	0.017
IMT (mm)	0.68 ± 0.13	0.87 ± 0.17	<0.001
ABPI (rel)	1.17 ± 0.11	1.11 ± 0.13	0.052

FMD-flow mediated dilation, IMT- intima-media thickness, ABPI- ankle brachial pressure index, p- for the difference between groups.

### Correlation analysis, multiple linear regression model and power analysis

**Figure 2:**
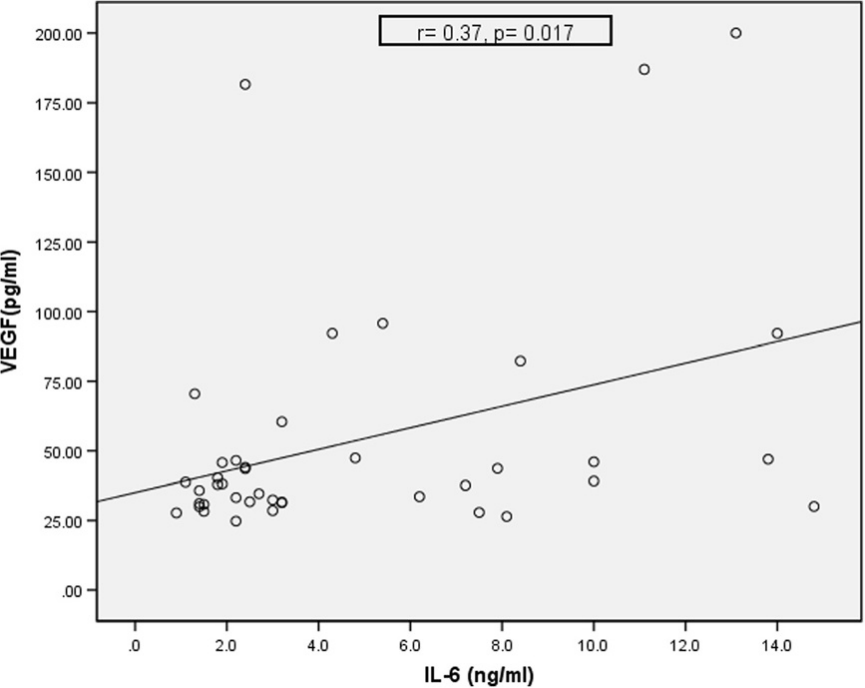
**Correlation between VEGF and IL-6 in patients.** *Legend: IL-6 - interleukin 6, VEGF- vascular endothelial growth factor, r - correlation coefficient, p - statistical significance.*

**Figure 3:**
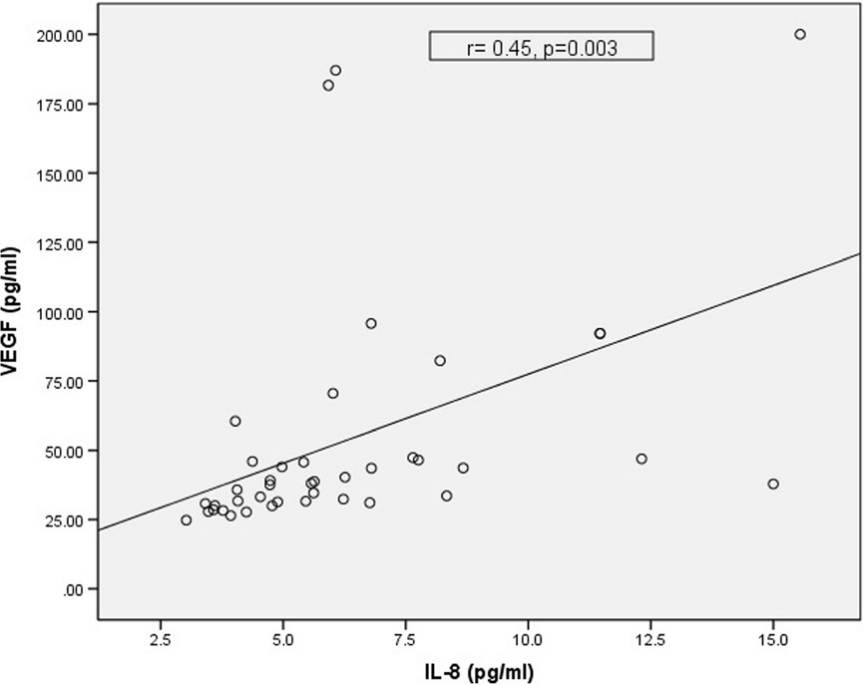
**Correlation between VEGF and IL-8 in patients.** *Legend: IL-8 - interleukin 8, VEGF- vascular endothelial growth factor, r - correlation coefficient, p - statistical significance.*

In the group of patients, a significant positive correlation was observed between VEGF and IL-6 (r = 0.37, p = 0.017) and between VEGF and IL-8 r = 0.45, p = 0.003. The correlations are plotted in Figures 2 and 3. No correlations between VEGF and parameters of atherosclerotic burden were obtained.

A multiple linear regression model was constructed to predict VEGF in the patients on the basis of classical coronary risk factors, inflammatory parameters and functional and structural characteristics of the arterial wall. Only factors that were significant in the univariant analysis were included in the model (IL-6 and IL-8). The adjusted R^2^ of this model was 0.226, with a high significance (p = 0.008). After adjustment only IL-6 (β = 0.338, p = 0.025) proved to be an independent determinant of VEGF.

We performed post-hoc power and sample size analysis for comparison between the controls and the patients regarding the level of VEGF, which was the main discovery and was found to be significant in our study. The group sample sizes of 25 and 41 achieve a 74% power to detect a difference of 17.6 between the null hypothesis that the both group means are 53.9 and the alternative hypothesis that the mean of group two is 36.3, with known group standard deviations of 42.7 and 8.9 and with a significance level (alpha) *a* = 0.05 using a two-sided two-sample t-test.

## Discussion

The aim of the present study was to explore the role of VEGF in the stable phase of coronary artery disease. We found that VEGF was increased in post-MI patients and furthermore that VEGF values correlated with inflammatory cytokines (IL-6 and IL-8), but not with parameters associated with the extent of the atherosclerotic burden. These results suggest that VEGF in the stable phase after MI is a part of the inflammatory response and very likely contributes to worsening of atherosclerosis by inducing plaque instability. If VEGF were to be involved in neovascularization as a compensatory mechanism to ischemia, it would certainly correlate with parameters of the atherosclerotic burden, but that was not the case.

It has been shown in clinical trials and animal models that VEGF promotes the development of collateral blood vessels in response to hypoxia [3, 12–14]. All our patients were in the acute phase of MI, successfully vascularized (percutaneous trans-luminal angioplasty), and at the time of the trial were asymptomatic, stable and without symptoms of angina pectoris. It is therefore interesting that they had increased VEGF levels. Although only a few studies have explored VEGF in patients with coronary artery disease, almost all were performed to determine the role of VEGF in the acute phase of MI where increased levels of VEGF were found. In one study the peak level for VEGF was reached during acute MI. After successful recanalization, the serum levels of VEGF rapidly (within 20–30 minutes) returned almost completely to the normal control range [15]. Other studies showed that VEGF returns to normal values after about three weeks or after 6 months [9, 10]. The differences in VEGF normalization time are most probably the result of different methods of treating AMI (PCI, or conservative treatment with heparin, which can reduce serum VEGF), as well as different study populations. There is no data for a period longer than 6 months.

Thus we explored the values of VEGF in patients on average 20.5 months after MI and found them to be increased, which is a new result. However, its explanation is quite complex. Hence, to clarify this we investigated the associations of VEGF with both inflammatory molecules and the atherosclerotic burden, and studied the levels of angiogenin, another angiogenic factor. The values of angiogenin were similar in the patients and controls and did not follow the increase of VEGF, thereby confirming the pivotal role of VEGF as an angiogenic factor. Furthermore, to obtain an appropriate answer to the basic question of the role of VEGF we analysed a very broad range of both inflammatory molecules and parameters of the atherosclerotic burden.

Atherosclerosis is a chronic inflammatory disease. In this context, we were interested whether VEGF is related to a possible on-going inflammation process. Namely, VEGF is believed to be a key molecule that connects angiogenesis with inflammatory response. In the context of this study the connection between angiogenesis and the intravascular arterial wall is of great interest. Therefore, the intensity of arterial wall inflammation was measured by the serum levels of different pro-inflammatory markers (IL-8, IL-6, hsCRP, VCAM1, ICAM1, p-selectin, e-selectin and TNFα). Importantly, in comparison to the controls, patients had significantly increased levels of IL-6 and hsCRP, whereas the values of the other inflammatory molecules were not elevated. Thus, one can conclude that IL-6 and hsCRP are the best markers of on-going arterial wall inflammation in the stable phase after MI. On evaluating the existence of possible associations, we found significant positive correlations between VEGF and IL-6, and between VEGF and IL-8. Thus, these results form a strong basis for stating that serum VEGF levels are related to the intensity of inflammation. This statement is also confirmed by the model of linear regression in which IL-6 proved to be an independent determinant of VEGF. We found no data in the literature on a correlation between VEGF and IL-6 and IL-8 in coronary patients. There are only a few studies describing a correlation between VEGF and different proinflammatory molecules in patients with coronary artery disease, and even then only in the acute phase. A positive correlation between VEGF and hsCRP was found in patients with acute MI [9, 16]. But to the best of our knowledge no studies describe a correlation between serum VEGF and IL-6 and IL-8, which has not been examined in the stable period after MI.

We also explored whether serum VEGF levels were related to parameters of the atherosclerotic burden, but did not find any relation. Notably, we did not find a correlation between VEGF and the severity of coronary artery disease, classified as one-, two- or three-vessel coronary artery disease found on the coronarograph. There are only some small and conflicting studies dealing with VEGF and the extent of coronary artery disease. Some studies found no correlation between serum VEGF levels and the severity (extent) of coronary atherosclerosis in patients measured on the 7th day after MI, in patients with stable AP and in patients with chest pain undergoing coronary angiography [17–19]. On the other hand, in a small Polish study, serum VEGF levels were higher in AMI patients with multi-vessel disease than in patients with single-vessel disease [16]. Larger studies are needed to clarify this relationship. Similarly, we found no correlation between IMT, measured on the common carotid artery, and serum VEGF levels. There are very few data about the association between IMT and VEGF in patients with coronary artery disease. In a small study, the authors found no differences in VEGF levels among patients with stable coronary artery disease and increased (above 1 mm) or normal IMT (below 1 mm) [20]. In the present study, we explored whether plasma VEGF levels were related to endothelial function, but found no significant correlation between FMD and VEGF levels. Data on the association of VEGF with vascular function is again limited. In vitro studies showed that VEGF is a potent survival factor for endothelial cells [21, 22]. It was shown that VEGF gene therapy may improve endothelial dysfunction in patients with end-stage coronary artery disease [9].

It is well-known that biomarkers of acute myocardial ischemia (acute coronary syndrome) are of significant clinical value and already in clinical use. The following markers for acute coronary syndrome are FDA-approved: creatin kinase, myoglobin and cardiac troponin. All three have become the gold standard for confirmation of acute coronary syndrome. Several other possible biomarkers are currently under investigation. However, in contrast, no clinically useful biomarker for prediction of recurrent myocardial infarction is currently available. Such a marker would undoubtedly be very valuable.

Taking all the results together we found that VEGF values are increased in the stable phase after MI and correlate with inflammatory markers but not with parameters of the atherosclerotic burden. Therefore, it appears logical that VEGF, driven by the inflammatory process, is involved in the on-going atherosclerosis process, probably inducing destabilization of coronary plaques by their neovascularization. On the other hand, the possibility that increased VEGF levels induce neovascularization in myocardium is much less probable since VEGF values did not correlate with the parameters of the atherosclerotic (i.e. ischemic) burden.

The limitations of the present study are that it was cross-sectional and not prospective and had a relatively small sample size. However the power of the study was quite large (74%) and the mentioned limitation did not affect the main finding of the study, which is increased VEGF levels in young male patients in the stable phase after MI.

## Conclusions

Several biomarkers that predict the clinical prognosis of patients with acute AMI have been identified. The relevance of our study is in using VEGF as an additional cardiovascular biomarker in the chronic stable phase of coronary artery disease. Conflicting data exists about the prognostic significance of VEGF levels in the acute phase after MI, but there is no data about the prognostic significance of VEGF in the stable phase of coronary artery disease. According to our findings, VEGF can have an important negative prognostic value in the stable phase after MI. This mechanistic study should be followed up by an observational study to definitively clarify the role of VEGF as a prognostic marker in patients in the stable phase after MI.
